# Supplementary material for: Thermally activated triplet exciton release for highly efficient tri-mode organic afterglow
Source: Nat Commun. 2020 Feb 12;11:842. doi: 10.1038/s41467-020-14669-3 (PMC7016145; doi:10.1038/s41467-020-14669-3)
Supplement: Supplementary file 1 — Supplementary Information [file 41467_2020_14669_MOESM1_ESM.pdf]

Supplementary Information

Thermally Activated Triplet Exciton Release for Highly Efficient Tri-mode Organic Afterglow

Jin et al.

1. Background information

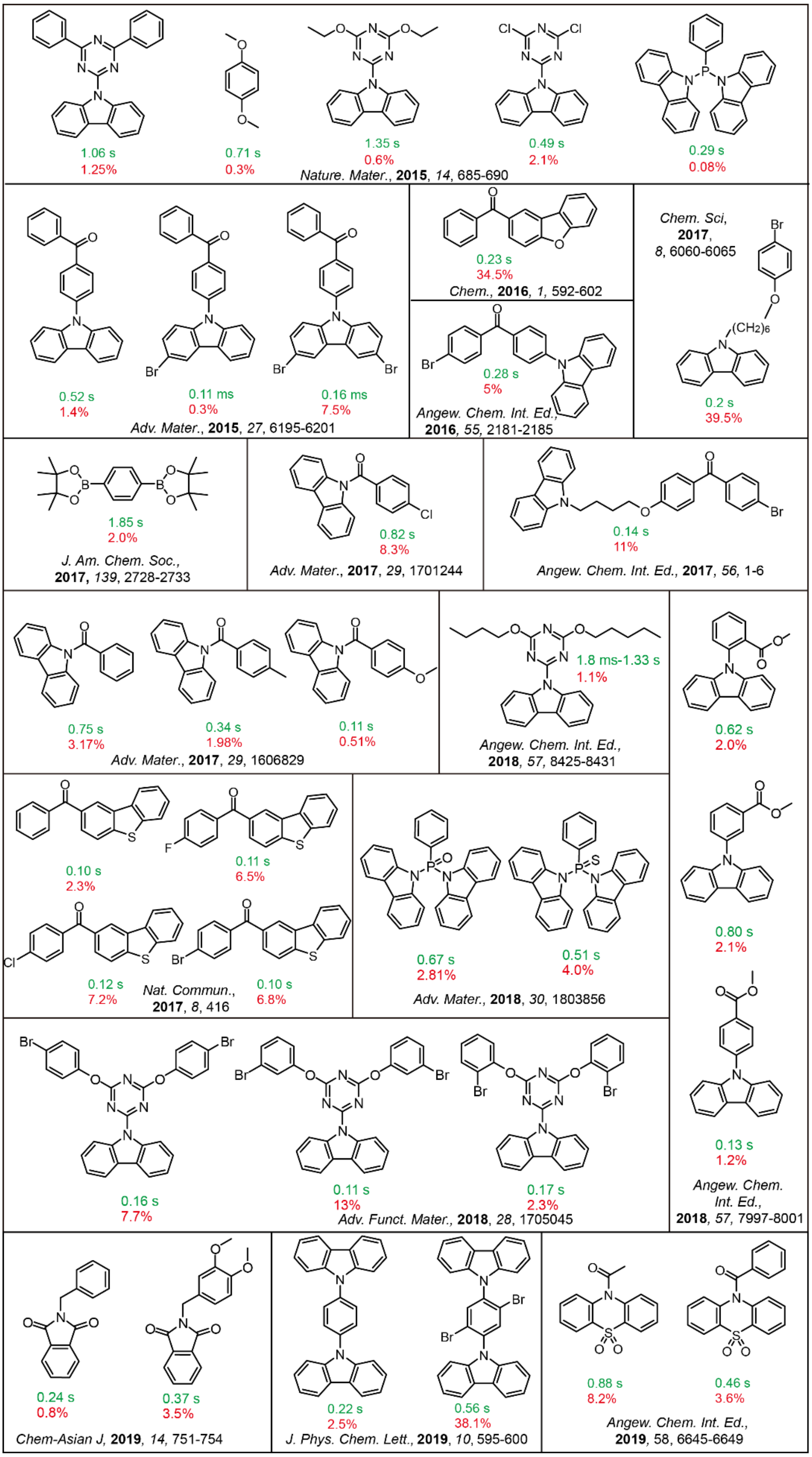

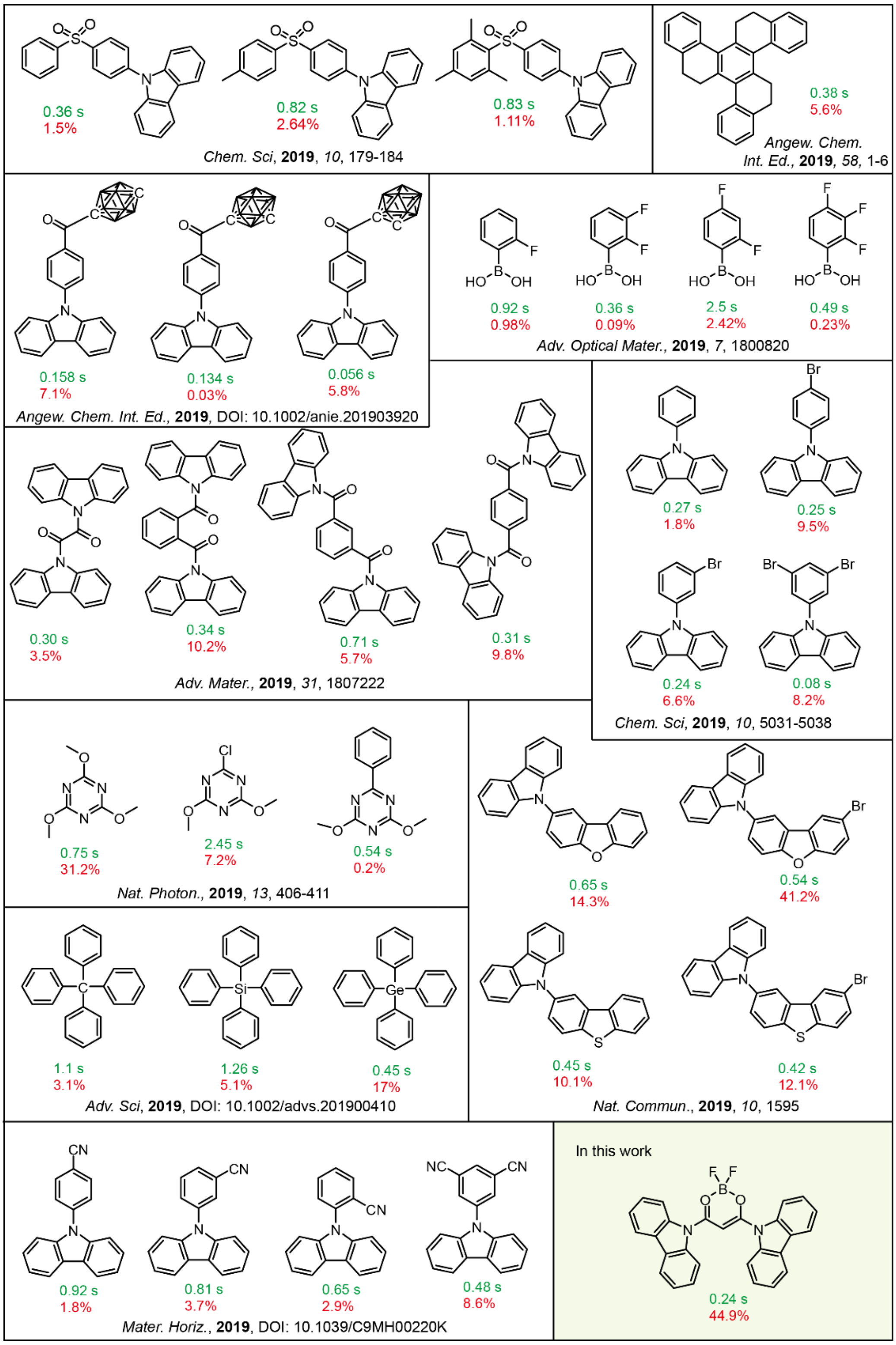

Supplementary Figure 1. The reported high-performance single-component organic afterglow materials with corresponding lifetimes (green) and afterglow efficiencies (red) at room temperature[1-27].

2. Synthesis and characterization

Materials

All reagents used in the experiments, unless otherwise specified, were purchased from Aldrich, Acros, or Alfa Aesar, and used without further purification. Manipulations involving air-sensitive reagents were performed in an atmosphere of dry argon. Toluene was dried and purified by sodium through distillation with benzophenone as a chromogenic reagent. The final product was purified by column chromatography and recrystallization from dichloromethane (CH_2Cl_2) and petroleum ether (PE) for four times, and was fully characterized by ^{1}H and ^{13}C-nuclear magnetic resonance (NMR), high-resolution mass spectra (HRMS) and single-crystal X-ray diffraction.

Instruments

^{1}H NMR and ^{13}C NMR spectra were recorded on a Bruker Ultra Shield Plus 400 MHz instruments with $CDCl_3$ as the solvents and tetramethylsilane (TMS) as the internal standard. HRMS was collected by a LCT Premier XE (Waters) HRMS spectrometry.

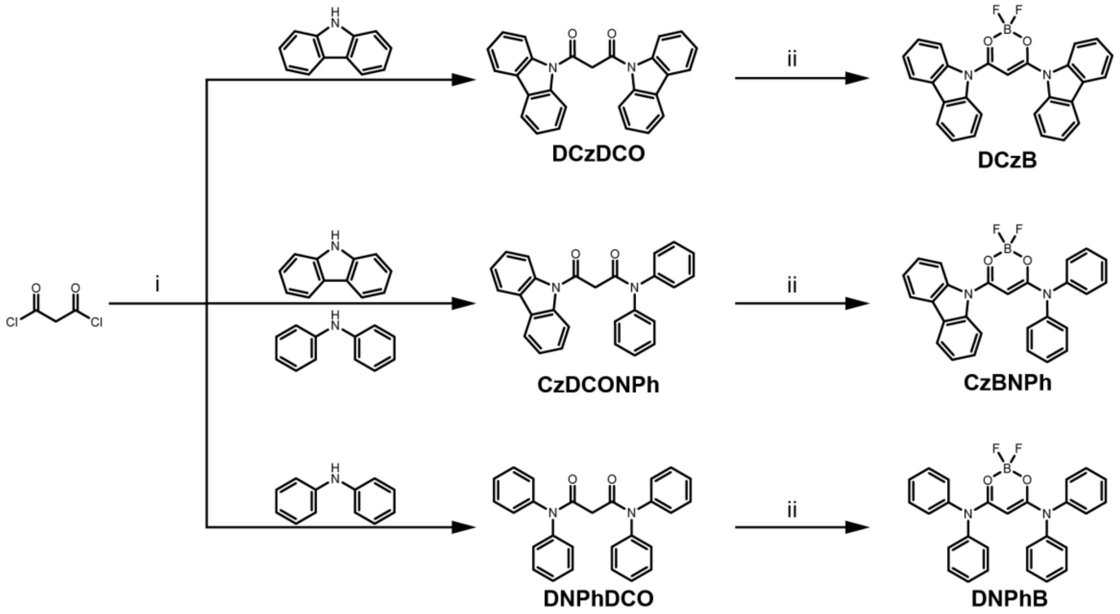

Supplementary Scheme 1. The synthetic route to prepare **DCzB**, **CzBNPh and DNPhB.** The reaction conditions: (i) toluene, room temperature; (ii) $BF_3{\cdot}Et_2O$, CH_2Cl_2, room temperature.

1, 3-Di(9*H*-carbazol-9-yl)propane-1, 3-dione (DCzDCO)

To a 50 mL round-bottom flask charged with 9H-carbazole (1.00 g, 6.0 mmol) was injected 30 mL dry toluene using a syringe under an argon atmosphere. Then, the malonyl dichloride (0.29 mL, 3.0 mmol) was injected to the toluene solution slowly. After stirring at room temperature for 5 min, the reaction was finished and the resulting **DCzDCO** in white precipitates was collected by filtration under reduced pressure. The precipitates were washed with sufficient acetone to remove the unreacted carbazole to obtain the purified **DCzDCO** for the next reaction. Yield: 0.90 g of white powder (75%).

Difluoroboron 1, 3-Di(9*H*-carbazol-9-yl)propane-1, 3-dione (DCzB)

To a solution of **DCzDCO** (0.50 g, 1.2 mmol) in 40 mL CH_2Cl_2 was added $BF_3{\cdot}Et_2O$ (0.46 mL, 3.6 mmol) under a dry argon atmosphere. The mixture was refluxed overnight. Then, the reaction was quenched by cooling to room temperature and washing with water (30 mL). The reaction mixture was extracted with CH_2Cl_2 (3×30 mL) and the organic layers were collected and dried with anhydrous Na_2SO_4. After removing the solvent by vacuum-rotary evaporation, the solid residue was purified by column chromatography (silica gel, 3:1 v/v, PE/CH_2Cl_2). Yield: 0.35 g of green powder (65%). ^1H NMR (400 MHz, $CDCl_3$): δ= 8.24 (d, J = 8.0 Hz, 4H), 8.05 (d, J = 8.0 Hz, 4H), 7.57 (t, J = 16 Hz, 4H), 7.48 (t, J = 16 Hz, 4H), 6.79 (s, 1H). ^{13}C NMR (100 MHz, $CDCl_3$): δ= 167.71, 137.86, 127.88, 127.22, 125.13, 120.57, 115.89, 80.27. HRMS (ESI): *m/z* calcd. for $C_{27}H_{18}BF_2N_2O_2$ ($[M+H]^+$), 451.1429; found, 451.1437.

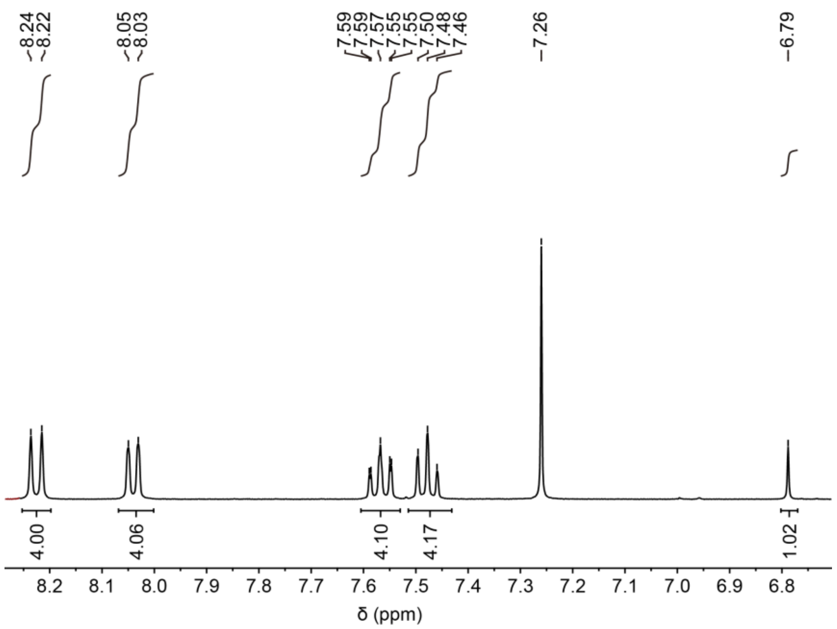

Supplementary Figure 2. ^1H NMR spectrum of **DCzB** in $CDCl_3$.

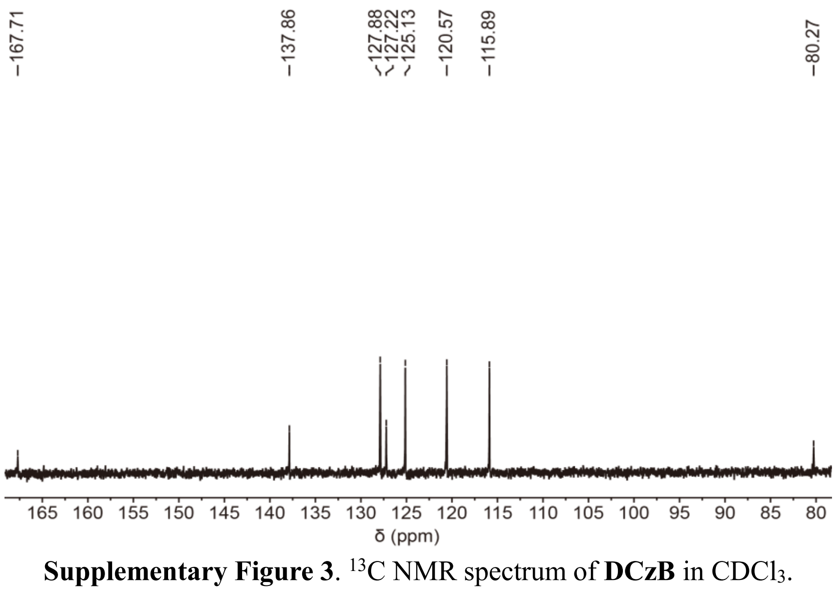

Supplementary Figure 3. ^{13}C NMR spectrum of **DCzB** in $CDCl_3$.

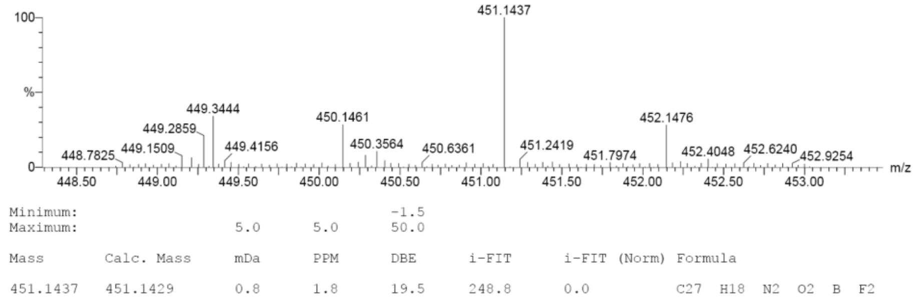

Supplementary Figure 4. HRMS of **DCzB**.

3-(9*H*-carbazol-9-yl)-3-oxo-N,N-diphenylpropanamide (CzDCONPh)

CzDCONPh was prepared under the identical synthetic conditions described in the preparation of **DCzDCO,** using 9H-carbazole (0.50 g, 3.0 mmol), diphenylamine (0.51 g, 3.0 mmol) and malonyl dichloride (0.29 mL, 3.0 mmol). Yield: 0.85 g of white powder (70%).

Difluoroboron 3-(9*H*-carbazol-9-yl)-3-oxo-N,N-diphenylpropanamide (CzBNPh)

CzBNPh was prepared under the identical synthetic conditions described in the preparation of **DCzB,** using **CzDCONPh** (0.50 g, 1.2 mmol), $BF_3{\cdot}Et_2O$ (0.46 mL, 3.6 mmol). Yield: 0.41 g of white powder (75%). ^{1}H NMR (400 MHz, *d*-DMSO, ppm): δ= 8.23 (d, J = 8.0 Hz, 2H), 7.87 (d, J = 8.0 Hz, 2H), 7.77 (d, J = 8.0 Hz, 2H), 7.66-7.40 (m, 12H), 5.34 (s, 1H). ^{13}C NMR (100 MHz, $CDCl_3$): δ= 169.3, 164.7, 138.0, 130.4, 129.5, 128.0, 127.1, 126.4, 126.3, 123.7, 120.1, 114.8, 77.6.

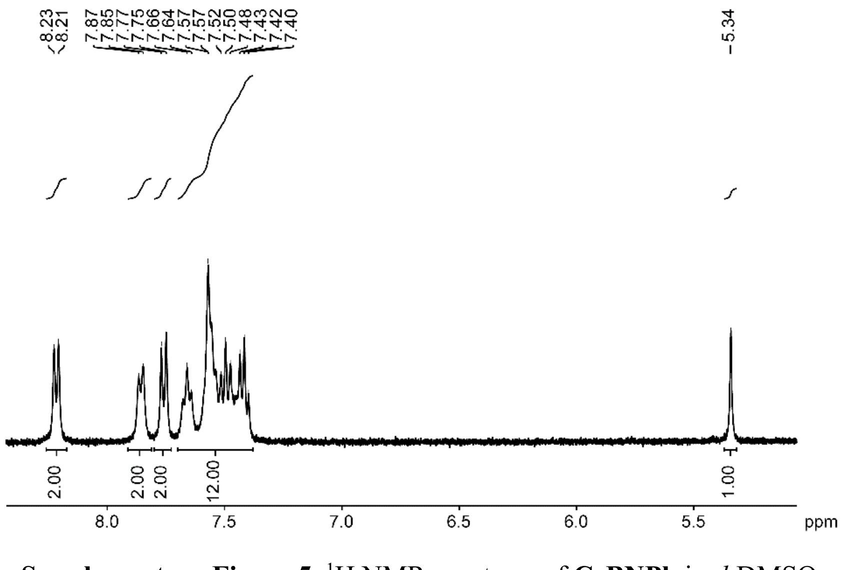

Supplementary Figure 5. ^{1}H NMR spectrum of **CzBNPh** in *d*-DMSO.

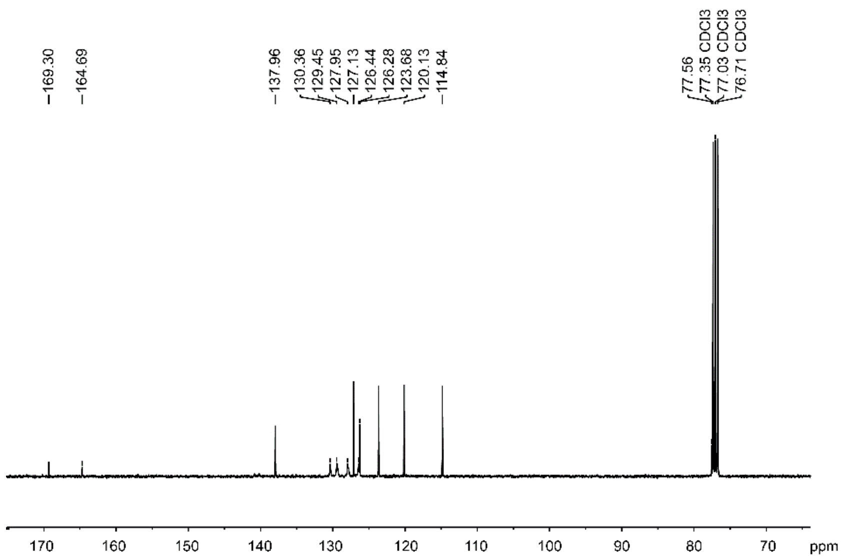

Supplementary Figure 6. ^{13}C NMR spectrum of **CzBNPh** in $CDCl_3$.

(Z)-3-(diphenylamino)-3-hydroxy-N,N-diphenylacrylamide (DNPhDCO)

DNPhDCO was prepared under the identical synthetic conditions described in the preparation of **DCzDCO,** using 9H-carbazole (0.50 g, 3.0 mmol), diphenylamine (0.51 g, 3.0 mmol) and malonyl dichloride (0.29 mL, 3.0 mmol). Yield: 0.98 g of white powder (80%).

Difluoroboron (Z)-3-(diphenylamino)-3-hydroxy-N,N-diphenylacrylamide (DNPhB)

DNPhB was prepared under the identical synthetic conditions described in the preparation of **DCzB,** using **CzDCONPh** (0.50 g, 1.2 mmol), $BF_3{\cdot}Et_2O$ (0.46 mL, 3.6 mmol). Yield: 0.38 g of white powder (70%). ^1H NMR (400 MHz, *d*-DMSO, ppm): δ= 7.40 (t, J = 16 Hz, 8H), 7.35-7.27 (m, 12H), 4.03 (s, 1H). ^{13}C NMR (100 MHz, $CDCl_3$): δ= 167.6, 141.2, 129.3, 127.3, 127.2, 71.3.

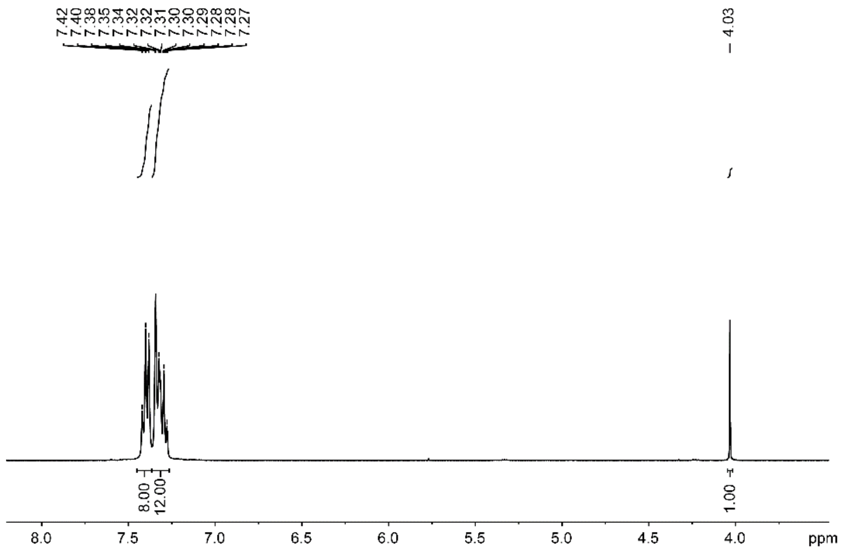

Supplementary Figure 7. ^1H NMR spectrum of **DNPhB** in *d*-DMSO.

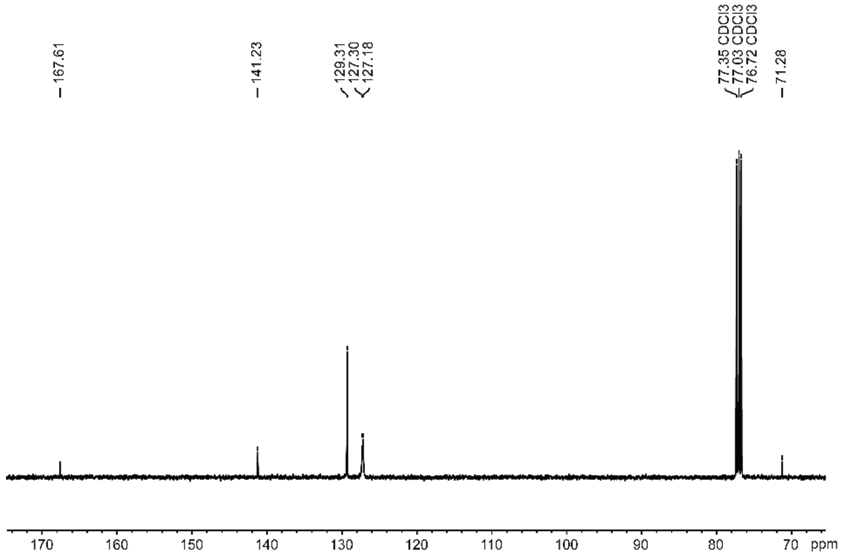

Supplementary Figure 8. ^{13}C NMR spectrum of **DNPhB** in $CDCl_3$.

3. Single crystal analysis

DCzB exhibits good solubility in common organic solvents; thus, highly pure material was obtained by column chromatography and its single crystals can be grown by slow evaporation of a mixed CH_2Cl_2 and PE solution at room temperature. The data of the single crystal structure were collected on a Bruker SMART APEX (II)-CCD at 298 K. Crystal structures were analyzed by Mercury 1.4 software. The CCDC number for **DCzB** is 1941925. The crystallographic data were summarized in Supplementary Table 1.

Supplementary Table 1. Crystallographic data of **DCzB**.

Empirical formula	$C_{27}H_{17}BF_2N_2O_2$
Formula weight (g mol^{-1})	450.23
Crystal color	green
Wavelength (Å)	0.71073
Crystal system	Orthorhombic
Space group	*Pnma*
a (Å)	14.382(16)
b (Å)	29.72(3)
c (Å)	5.070(5)
α (deg)	90
β (deg)	90
γ (deg)	90
V (Å^3)	2167(4)
Z	4
Density (g cm^{-3})	1.38
μ (mm^{-1})	0.099
T_{min}, T_{max}	0.986, 0.990
F(000)	928
h_{max}, k_{max}, l_{max}	14,38,6
$Theta_{max}$	28.965
CCDC number	1941925

4. Thermal properties

Thermogravimetric analysis (TGA) was conducted on a DTG-60 Shimadzu thermal analyst system under a heating rate of 10ºC/min and a nitrogen flow rate of 50 cm^3/min. TGA reveals that **DCzB** has a good thermal stability among boron-containing compounds with a high decomposition temperature (T_d = 251°C), owing to its stable four-coordinate boron structure[28]. Melting point of 220°C is obtained from melting point apparatus. Thermogravimetric analysis reveals that this D-A-D molecule has a decomposition temperature over 250°C, which is high thermal stabilities among the boron-containing optoelectronic materials[28,29].

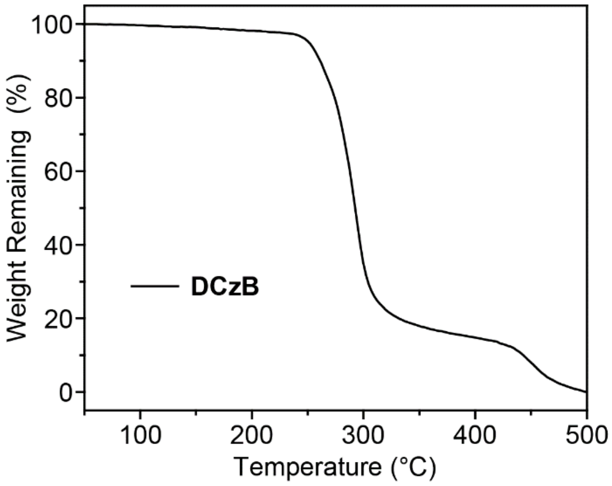

Supplementary Figure 9. TGA curve of **DCzB**.

5. Photophysical properties

Ultraviolet/visible (UV/Vis) and photoluminescence (PL) spectra of **DCzB** in dilute toluene, CH_2Cl_2, tetrahydrofuran (THF), trichloromethane ($CHCl_3$) and solid film were measured on a Lambda 650 S Perkin Elmer UV/VIS spectrophotometer and Edinburgh FLS 980 fluorescence spectrophotometer, respectively. For fluorescence lifetime measurements, a picosecond pulsed light emitting diode (EPLED-380, wavelength: 377 nm; pulse width: 947.7 ps) was used. The phosphorescence spectrum of **DCzB** in dilute toluene was obtained using an Edinburgh FLS 980 fluorescence spectrophotometer at 77 K in a dewar vessel with 5 ms delay time after excitation using a microsecond (μs) flash lamp. The microsecond flash lamp produces short, typically a few microsecond, and high irradiance optical pulses for room temperature phosphorescence measurements in the range from microseconds to seconds. The afterglow spectra, kinetic measurements, lifetime (τ) and time-resolved emission spectra of **DCzB** crystal were also measured on an Edinburgh FLS 980 fluorescence spectrophotometer. The absolute photoluminescence quantum yield (PLQY) was obtained using an integrating sphere. The lifetimes of the luminescence were figured out by fitting the luminescent intensity decay curve (I(t)) with a multi-exponential decay function in Supplementary Equation (1):

$$I(t) = \sum_i A_i e^{-\frac{t}{\tau_i}} \quad (1)$$

where A_i and τ_i represent the amplitudes and lifetimes of the individual PL components in multi-exponential decay profiles, respectively. The temperature-dependent afterglow spectra and lifetime decay profiles were measured using a closed cycle cryostat (oxford-instruments Optistat DN2). The photographs were taken by a Nikon D90 camera.

5.1 Absorption and PL properties at room temperature

Absorption and steady-state PL spectra of **DCzB** in different solvents (Supplementary Figure 10) exhibit a maximum absorption around at 380 nm, which should be ascribed to intermolecular charge transfer (ICT) transition from the donor of carbazole group to the acceptor of difluoroboron β-diketonate unit, since the absorption bands of both the donor and acceptor units are significantly blue-shifted (around 295 nm)[30]. This ICT characteristic can be confirmed by the remarkable positive solvatochromism in PL emission, showing gradually red-shifted PL peaks with the polarity increase of the solvents. Thus, **DCzB** should be a typical ICT type molecule due to its D–A electronic structure.

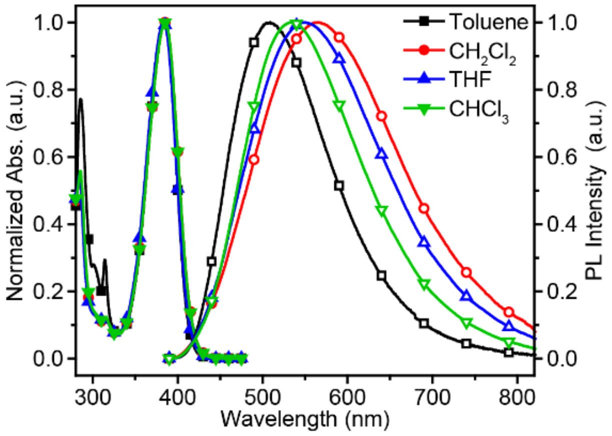

Supplementary Figure 10. UV-vis absorption (solid symbols) and photoluminescence spectra (open symbols) of **DCzB** in toluene, CH_2Cl_2, $CHCl_3$ and THF at room temperature.

5.2 Aggregation induced emission (AIE) property of DCzB

AIE properties of **DCzB** were observed in THF/water mixed solvents with different water fraction (f_w) (Supplementary Figure 11). **DCzB** exhibits weak emission in pure THF solution, but its PL enhances gradually with the increase of f_w, demonstrating a typical AIE characteristic. In addition, when the value of f_w increases, the PL lifetime also increases, suggesting a turn of fluorescence in THF to phosphorescence of aggregates at high f_w values. Therefore, **DCzB** also has the aggregation induced phosphorescence (AIP) property (Supplementary Figure 12).

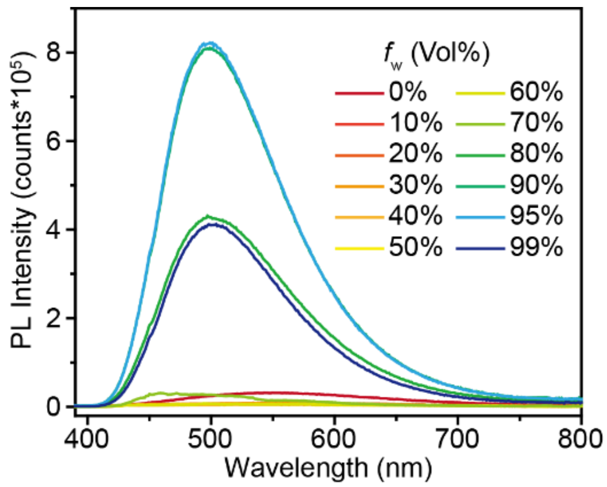

Supplementary Figure 11. Photoluminescence spectra of **DCzB** in THF/water mixtures (~10^{-5} mol/L) with different water fractions (f_W). The excitation wavelength is at 380 nm.

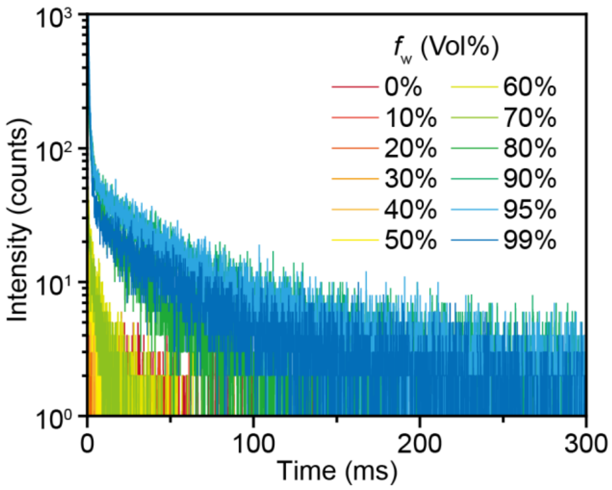

Supplementary Figure 12. Lifetime decay profiles of **DCzB** in THF/water mixtures with different water fraction (f_W). The excitation wavelength is at 380 nm.

5.3 Thermally activated delayed fluorescence (TADF) property of DCzB

By comparing the PL spectra of **DCzB** in aerated and argon-degassed toluene solutions, the PL enhances significantly in the absence of triplet-quenching oxygen (Supplementary Figure 13), indicating the emission is related to the triplet state. Through the fluorescence and phosphorescence spectra in dilute toluene at 77 K (Supplementary Figure 14a), the singlet-triplet splitting energy (ΔE_{ST}) were identified to be 0.15 eV, which is small enough for the typical TADF molecules. Nevertheless, efficient phosphorescent emission was also observed with lifetime of 398.65 ms in dilute solution at 77 K (Supplementary Figure 14b).

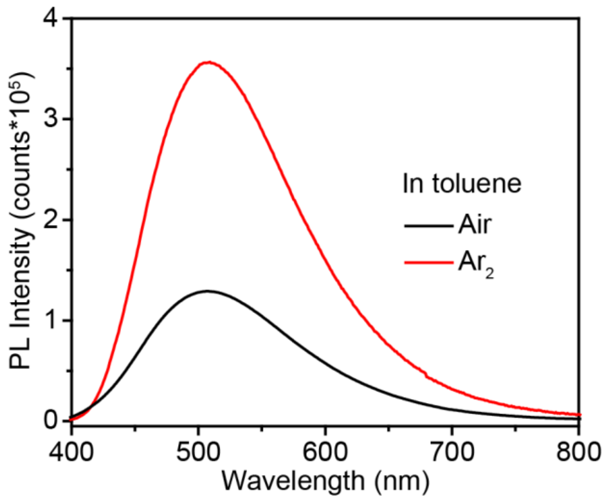

Supplementary Figure 13. PL spectra of **DCzB** in aerated and argon-degassed toluene solutions at room temperature. The excitation wavelength is at 380 nm.

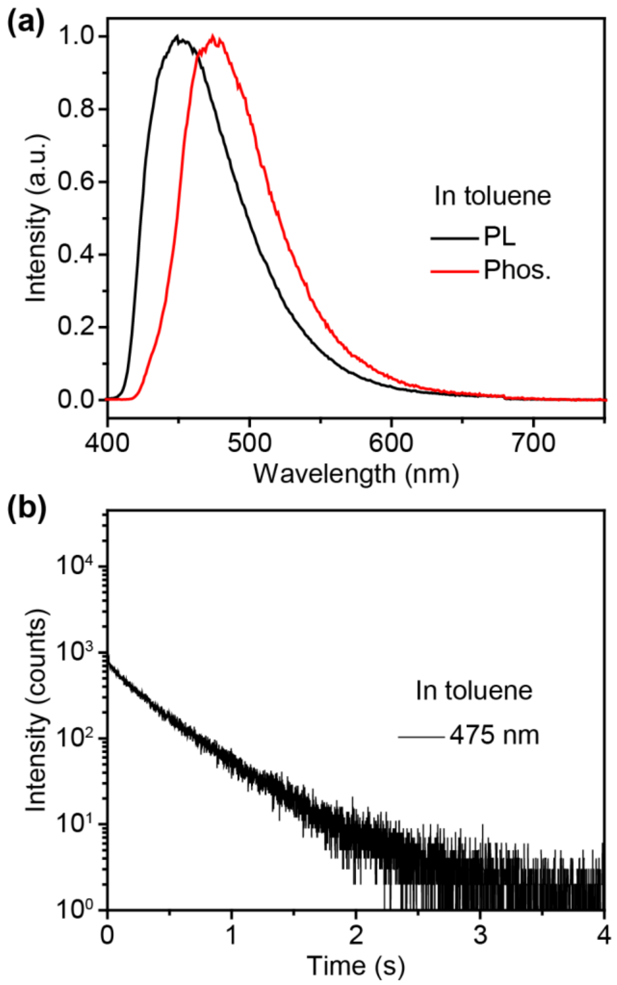

Supplementary Figure 14. (a) Steady state PL and phosphorescence spectra and (b) phosphorescence (475 nm) decay profile of **DCzB** in dilute toluene solution at 77 K. The excitation wavelength is at 380 nm.

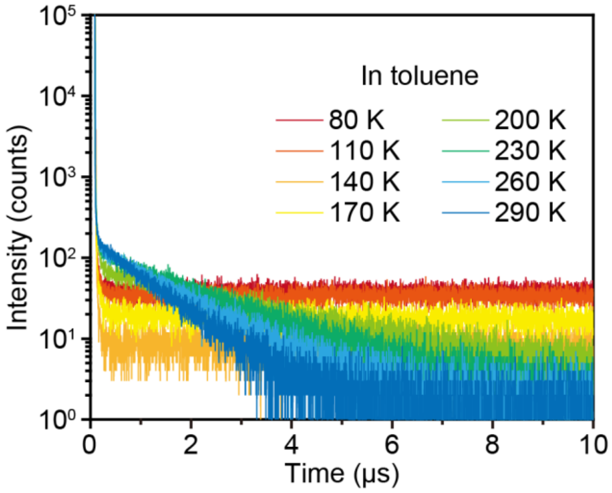

Supplementary Figure 15. Transient PL decay curves at different temperatures of **DCzB** in degassed toluene solution. The excitation wavelength is at 380 nm.

5.4 Afterglow properties of DCzB crystal

In crystal state, **DCzB** exhibits high PLQY of 55.0% and large afterglow component of 81.6% in avarage (Supplementary Table 2). Therefore, we can conclude that the afterglow efficiency of **DCzB** crystal is up to 44.9%. This efficiency is the highest afterglow efficiency among the single component organic afterglow molecules reported so far.

Supplementary Table 2. PL lifetimes of **DCzB** crystal excited at 380 nm under ambient conditions

Wavelength (nm)	τ_1 (ms)	A_1 (%)	τ_2 (ms)	A_2 (%)	χ^2
475	41.75	18.05	240.12	81.95	1.138
495	37.38	18.76	234.39	81.24	1.271
525	36.18	18.39	231.98	81.61	1.288

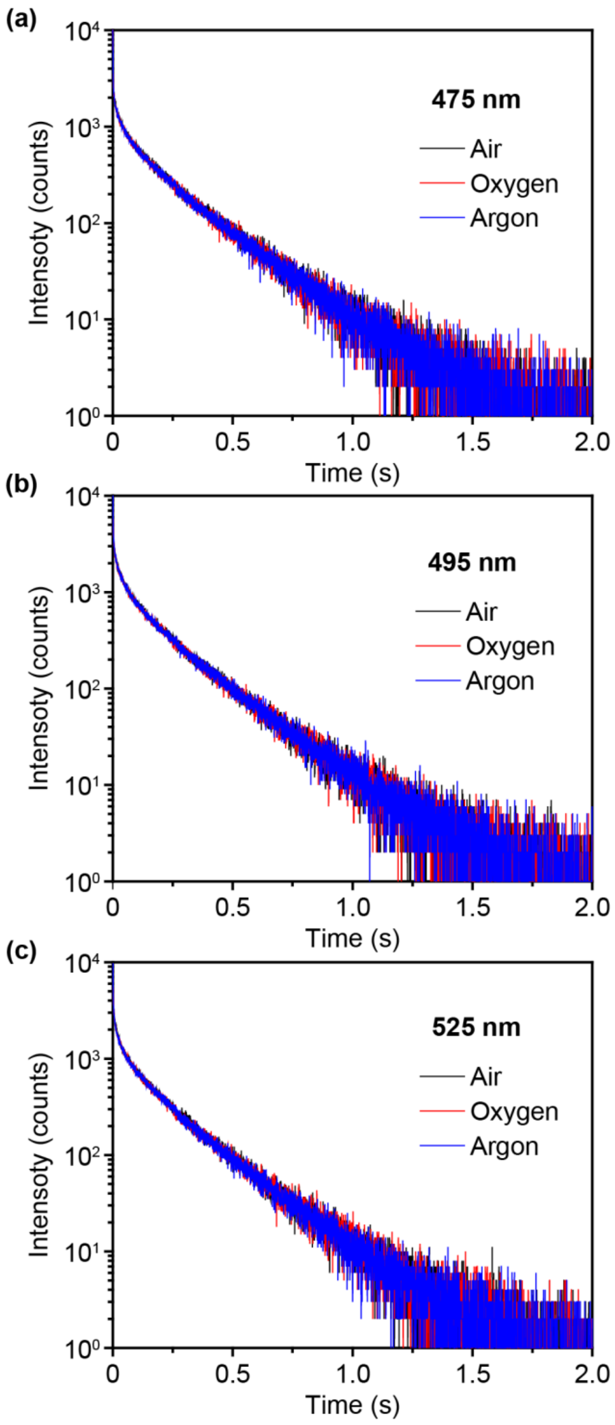

Supplementary Figure 16. Afterglow decay profiles of **DCzB** crystal at (a) 475 nm, (b) 495 nm and (c) 525 nm emission bands in different atmosphere of air, oxygen and argon at room temperature. The excitation wavelength is at 380 nm.

The influence of the excitation strength on afterglow emission was investigated by monitoring the afterglow emission intensity with different Iris of the excitation source at 380 nm. Iris is a parameter in Edinburgh FLS 980 spectrophotometer used to adjust the excitation light intensity. Larger Iris means the stronger excitation intensity. By increasing Iris from 10% to 100%, the excitation intensity can be controlled to increase linearly. From Supplementary Figures 17a-b, the

excitation light at 380 nm with varied Iris was turned on at 5 s, maintained for 10 s, and then turned off. The resultant afterglow emission intensity remains stable during the excitation for 10 s, then decays in exponential manner after the turn-off of the excitation, while the steady-state emission and afterglow luminescence intensity increases linearly. Moreover, the influences of the excitation duration on the afterglow emission were investigated by monitoring its emission band with different irradiating time of the excitation (Supplementary Figures 17c-d). The steady-state and afterglow emission intensities become almost constant when the excitation duration extends from 0.4 to 10 s, indicating that the afterglow can be effectively excited with low intensity and short duration of light under ambient conditions.

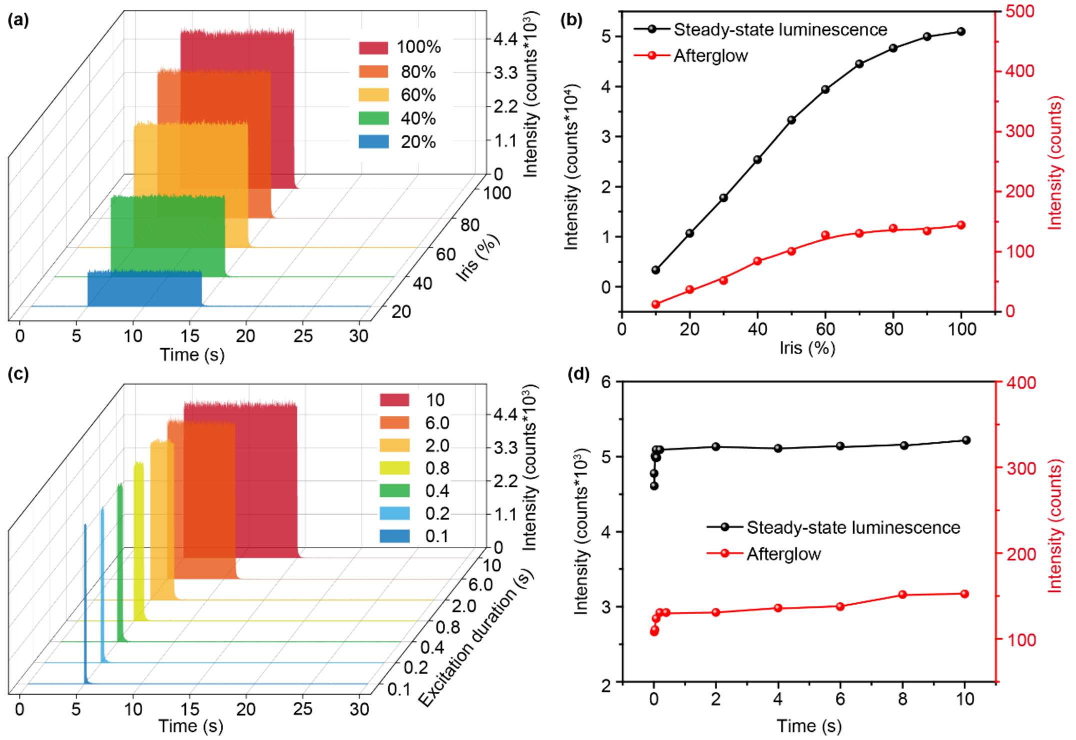

Supplementary Figure 17. (a) PL intensity profiles of 525 nm for **DCzB** crystal with corresponding steady-state (black) and afterglow (red) luminescence strengths (b) as a function of Iris (20, 40, 60, 80 and 100%) when excited at 380 nm under ambient conditions. (c) PL intensity profiles of 525 nm for **DCzB** crystal with corresponding steady-state (black) and afterglow (red) luminescence strengths (d) as a function of time upon excitation with different irradiating time (0.1, 0.2, 0.4, 0.8, 2.0, 6.0, 10 s) when excited at 380 nm under ambient conditions.

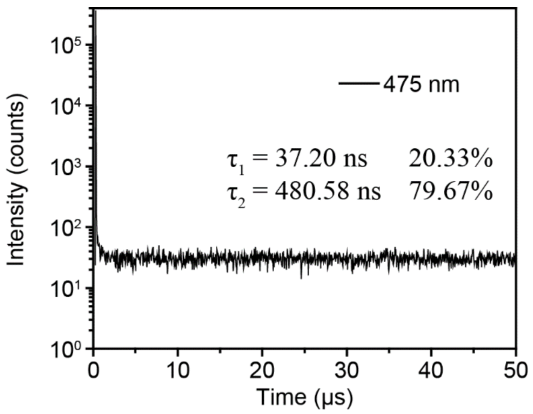

Supplementary Figure 18. Lifetime decay profile of the 475 nm emission band of **DCzB** crystal recorded at 300 K. The excitation wavelength is at 380 nm.

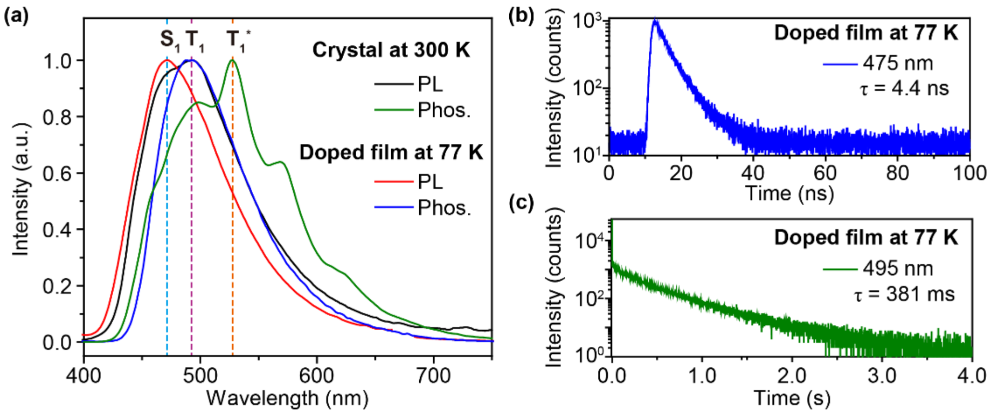

Supplementary Figure 19. (a) Photoluminescence (black and red curves) and phosphorescence (green and blue curves) spectra of DCzB crystal at 300 K and DCzB (5 wt%)-doped film in polymethyl methacrylate (PMMA) at 77 K. Lifetime decay curves at (b) 475 nm and (c) 495 nm of DCzB (5 wt%)-doped film in PMMA at 77 K.

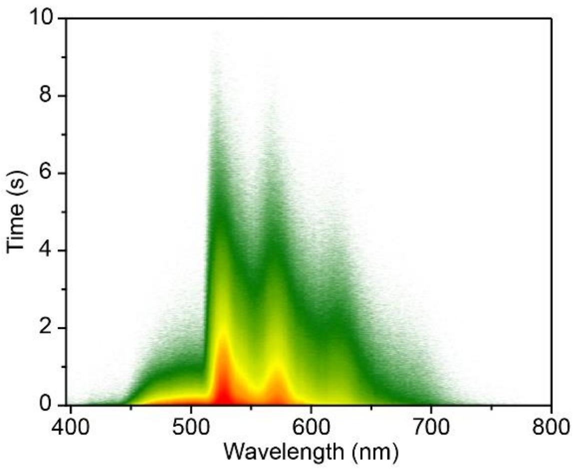

Supplementary Figure 20. Transient PL decay image of the **DCzB** crystal recorded at 77 K. The excitation wavelength is at 380 nm.

In order to uncover the mechanism of the thermally activated tri-mode afterglow, the temperature-dependent steady-state PL and afterglow emission were investigated by changing the temperature from 77 K to 300 K. The steady-state PL enhances with the reduced temperature owing to the suppressed nonradiative transition (Supplementary Figure 21). In afterglow decay profiles, due to the both suppressed nonradiative transition and thermally activated triplet exciton release processes, the lifetimes of 475 and 495 nm emission reduce firstly then elongate, when the temperature drops from 300 K to 77 K. This changing trend is in consistent with TADF, since these two emission peaks are corresponding to the fluorescence and phosphorescence (Supplementary Figures 22a-b)[31]. The afterglow lifetime of 525 nm emission is due to the radiative decay of the stable triplet excited state of T_1^*; thus, it rises gradually at reduced temperature owing to the suppressed nonradiative transition (Supplementary Figure 22c)[32]. Therefore, these measurements give clear evidences for that the tri-mode afterglow is realized by populating S_1 and T_1 with the long-lifetime excitons on T_1^* through thermally activated exciton release and reverse intersystem crossing processes.

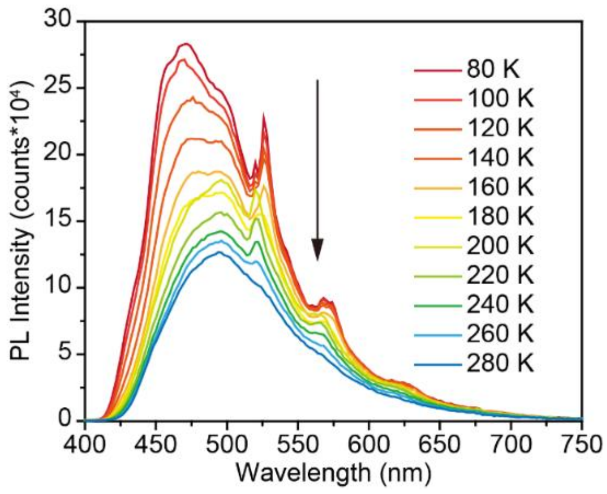

Supplementary Figure 21. Temperature-dependent photoluminescence spectra of **DCzB** crystal at different temperatures from 80 K to 280 K. The excitation wavelength is at 380 nm.

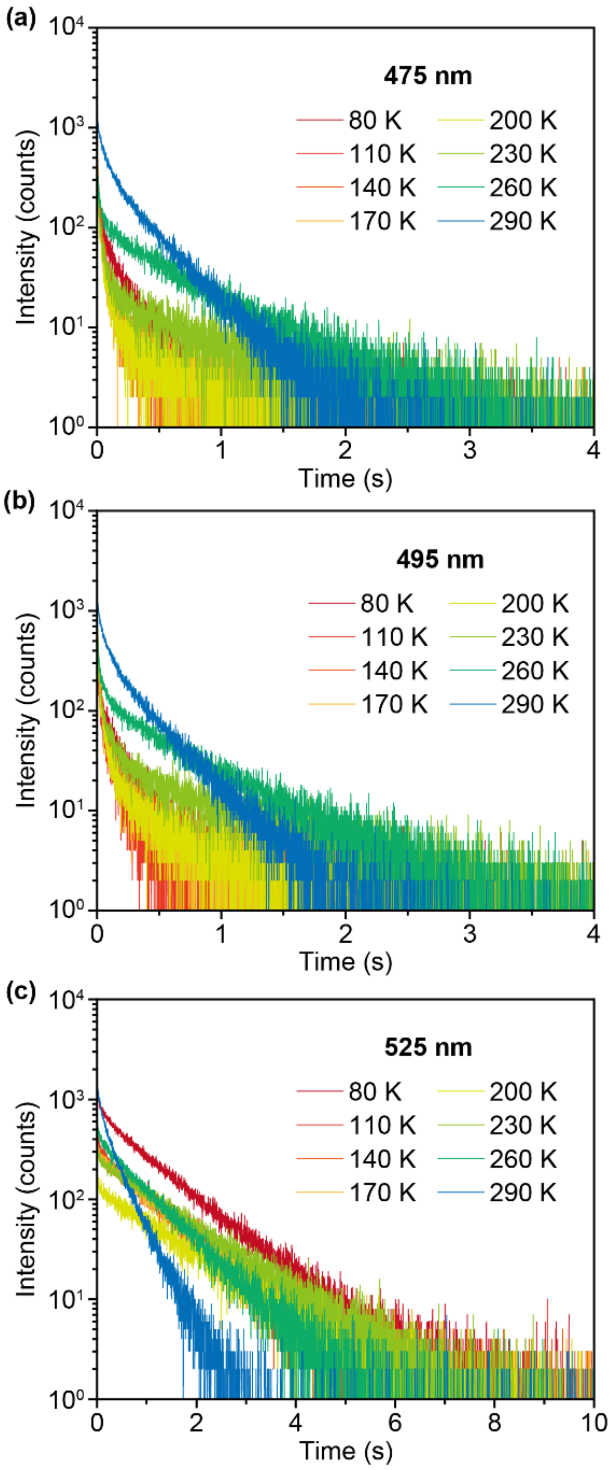

Supplementary Figure 22. Temperature-dependent afterglow decay profiles at (a) 475, (b) 495 and (c) 525 nm emission bands of **DCzB** crystal. The excitation wavelength is at 380 nm.

6. Theoretical calculations

Density functional theory (DFT) and time-dependent DFT (TD-DFT) computations were carried out using Gaussian 09 package. The ground state geometry was optimized by DFT method of B3LYP/6-31G(d); the optimized stationary point was further characterized by harmonic vibrational frequency analysis to ensure that real local minima were reached. TD-DFT calculations were performed to predict the excitation energies in the *n*-th singlet (S_n) and *n*-th triplet (T_n) states on the basis of the optimized ground structure via spin-restricted formalism using B3LYP/6-31G(d) (Supplementary Table 3). Spin-orbit coupling (SOC) matrix elements between the singlet excited states and triplet excited states were calculated with quadratic response function methods using the Dalton program[34,35]. The SOC of **DCzB** was performed at the optimized geometry of the lowest triplet excited state (T_1) using B3LYP functional and cc-PVTZ basis set (Supplementary Table 4).

The quantum mechanics/molecular mechanics (QM/MM) model was built from the single crystal structure and was implemented to evaluate the electronic properties of the active QM molecule embedded in the aggregated crystal state, while the surrounding molecules were defined as rigid MM part to model the effect of solid-state environment. The high layer for QM is calculated by the TD-DFT method of B3LYP/6-31G(d), and the low layer for MM is described by the universal force field (UFF) enhanced by Coulomb interactions which are in-line with the quantum method. The dimer was set up to high layer for QM calculated by the TD-DFT method of B3LYP/6-31G(d). The longer π-π interaction distance on the excited states compared to that on the ground state was observed, suggesting that photoexcitation can disturb the π-π interactions significantly.

In the ground state, the dihedral angle between donor and acceptor reduces from 32.6˚ in single molecular state to 19.9˚ in crystal, which may be related to the enhanced local-excited (LE) state emission feature in film state. When **DCzB** was photo-excited to either S_1 or T_1 states, much larger torsion angles were resulted, suggesting that the photoexcitation may disturb the π-π stacking of the molecules for a shallow exciton trapping depth.

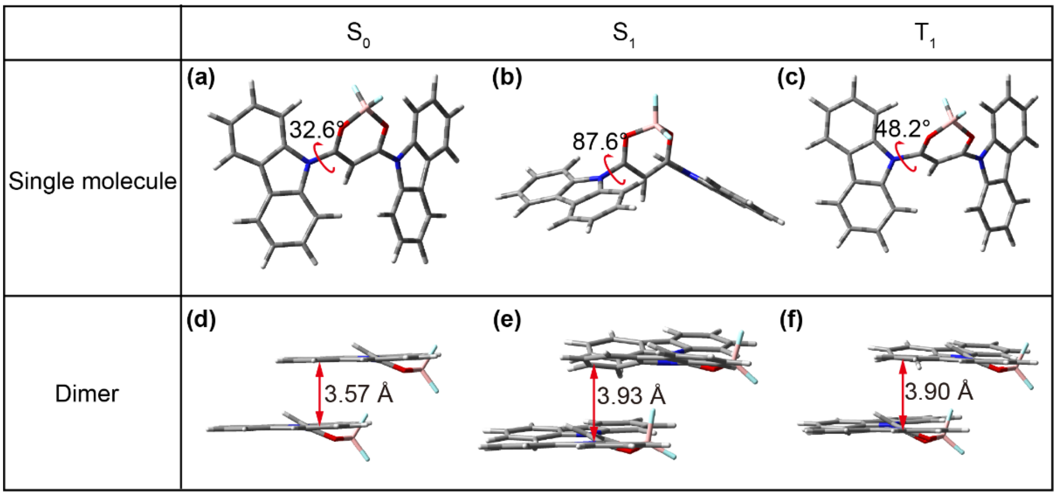

Supplementary Scheme 2. The dihedral angle between carbazole (donor) and difluoroboron β-diketonate (acceptor) of DCzB in the optimized single molecular geometry at (a) S_0, (b) S_1 and (c) T_1, respectively. The π-π stacking in (d) the single crystal and optimized dimer structures at (e) S_1 and (f) T_1 states.

Supplementary Table 3. TD-DFT calculated singlet and triplet excited states transition configurations of **DCzB**.

Excited state	*n*-th	Energy (eV)	Transition configuration (%)
S_n	1	3.359	H ->L (99.09)
	2	3.5237	H-1 ->L (99.00)
	3	3.5241	H-2 ->L (99.06)
	4	3.8522	H-3 ->L (98.52)
	5	4.2758	H-3 ->L+2 (6.45) H-2 ->L+3 (2.42) H-1 ->L+4 (3.27) H ->L+1 (84.86)
	6	4.3124	H-3 ->L+1 (9.97) H-2 ->L+4 (3.40) H-1 ->L+3 (2.55) H ->L+2 (80.96)
	7	4.4824	H-4 ->L (78.63) H-2 ->L+1 (10.41) H-1 ->L+2 (8.13)
	8	4.486	H-5 ->L (77.05) H-2 ->L+2 (8.72) H-1 ->L+1 (11.08)
	9	4.7128	H-5 ->L (2.65) H-3 ->L+1 (65.08) H-2 ->L+4 (2.19) H-1 ->L+1 (10.06) H-1 ->L+3 (2.04) H ->L+2 (13.01)
	10	4.7338	H-5 ->L (13.78) H-3 ->L+1 (13.26) H-1 ->L+1 (56.23) H ->L+4 (7.35)
T_n	1	2.83	H-6 ->L 5.102735 H ->L 91.57375
	2	3.0719	H-3 ->L+3 (2.32) H-2 ->L (33.24) H-2 ->L+2 (21.43) H-1 ->L+1 (26.53) H ->L+4 (3.84)
	3	3.072	H-3 ->L+4 3.508601 H-2 ->L+1 26.30155 H-1 ->L (33.23) H-1 ->L+2 (21.62) H ->L+3 (2.54)
	4	3.1968	H-3 ->L (91.00)
	5	3.5242	H-2 ->L+1 (13.30) H-1 ->L (62.97) H-1 ->L+2 (15.20)
	6	3.5252	H-2 ->L (62.71) H-2 ->L+2 (15.04) H-1 ->L+1 (13.50)
	7	3.6767	H-6 ->L (12.36) H-5 ->L+4 (3.85) H-4 ->L+3 (3.18) H-3 ->L+1 (22.84) H-3 ->L+5 (2.73) H ->L+2 (43.64)
	8	3.6886	H-4 ->L+4 (2.36) H-3 ->L (2.05) H-3 ->L+2 (27.81) H ->L+1 (56.89)
	9	3.7735	H-6 ->L (67.54) H-3 ->L+1 (6.75) H ->L (4.18) H ->L+2 (11.80)
	10	4.1153	H-5 ->L+3 (2.03) H-5 ->L+5 (2.83) H-4 ->L (30.96) H-4 ->L+2 (3.03) H-3 ->L+4 (14.22) H-2 ->L+1 (8.93) H-1 ->L+2 (8.30) H ->L+3 (17.73) H ->L+5 (4.32)

Supplementary Table 4. Theoretically calculated S_1 -T_n energy gap and S_1/T_n spin-orbit coupling (SOC) constants of **DCzB**.

S_1/T_n	S_1/T_n energy gap	SOC
**S_1/T_1**	0.53	2.02
**S_1/T_2**	0.29	0.37
**S_1/T_3**	0.29	0.16
**S_1/T_4**	0.16	0.08
**S_1/T_5**	-0.17	0.90
**S_1/T_6**	-0.17	0.21
**S_1/T_7**	-0.32	2.84
**S_1/T_8**	-0.33	0.43
**S_1/T_9**	-0.41	0.29
**S_1/T_{10}**	-0.76	0.94

7. H-aggregation for stabilizing triplet excitons

H-aggregation has been proved to be critical for stabilizing triplet exciton, resulting the efficient OURTP emission at the solid states since it is highly effective in trapping the excited states in low-lying aggregation-split energy level[36]. To identify the existence of the H-aggregation, the single crystal structure of **DCzB** was investigated. According to the molecular exciton theory[37], the positive exciton splitting energy (Δε) of the selected molecular packing manifests the presence of H-aggregates in Supplementary Equation (2):

$$\Delta\varepsilon = \frac{2|M|^2}{r_{uv}^3}(\cos\alpha - 3\cos\theta_1\cos\theta_2) \quad (2)$$

where M is the electric dipole transition moment, r_{uv} represents the intermolecular distance between the molecular pair, α is the angle between the transition dipole moments of the two molecules, and θ_1 and θ_2 are angles between transition dipole moments of the two molecules and the interconnection of their molecular centers, respectively. Based on this criterion, large and positive $\Delta\varepsilon$ values for strong H-aggregation were observed in **DCzB**.

8. Applications

8.1 Bioimaging

Preparation of nanoparticles of DCzB: The **DCzB** nanoparticles in aqueous solution were prepared via a well-documented matrix-encapsulation method in a bottom-up approach (nanoprecipitation) with appropriate optimization. PEG-b-PPG-b-PEG (F127) was chosen as the encapsulation matrix due to its good encapsulation performance and high biocompatibility. Briefly, F127 (10 mg) and **DCzB** (1 mg) were dissolved into a THF (1 mL) solution. The mixture was then rapidly injected into Milli-Q water (10 mL) under continuous sonication in a sonicator bath (Branson) for 5 min. Then, THF was evaporated with a gentle nitrogen flow. Finally, the aqueous solution was filtered through a 0.22 μm PVDF syringe driven filter (Millipore). The obtained nanoparticle solution was stored in dark at 4 °C.

Characterization method: The particle size and morphology of the **DCzB** nanoparticles were characterized by dynamic light scattering (DLS) and transmission electron microscope (TEM). DLS was performed using a quartz cuvette at 25 °C. Average particle sizes were determined by laser light scattering with a particle size analyzer (90 Plus, Brookhaven Instruments Co., Holtsville, NY, USA) at a fixed angle of 90° at room temperature. Samples for TEM measurements were prepared by drop casting the **DCzB** nanoparticle dispersion onto copper grids. The samples were allowed to dry at room temperature, then the TEM images were obtained using a Hitachi H-H7500 microscope operated at 120 kV. The steady-state PL and phosphorescence spectra of **DCzB** nanoparticles were obtained on Edinburgh FLS 980 fluorescence spectrophotometer with xenon lamp and microsecond lash lamp. Confocal luminescence imaging was carried out on an Olympus FV1000 laser scanning confocal microscope equipped with a 40 immersion objective len. The HeLa cells were incubated with the **DCzB** nanoparticles (10 μM) for 3 h at 37 °C. Then the cells were washed with PBS (phosphate buffer saline) for three times and transferred into Live Cell Imaging System for confocal luminescence imaging. Five samples of thus treated cells were randomly selected for imaging tests and typical results were illustrated. Under the excitation of 405 nm semiconductor laser, the emission was collected from 450 to 550 nm. Phosphorescence lifetime imaging (PLIM) was carried out on the Olympus IX81 laser scanning confocal microscope. The photoluminescence signal was detected by the system of the confocal microscope and correlative calculation of the data was performed by professional software which was provided by PicoQuant Company. The light from the pulse diode laser head (PicoQuant, PDL 800-D) with excitation wavelength of 405 nm with a 40×/NA 0.95 objective lens for single-photon excitation. These experiments were repeated three times and similar results were observed.

8.2 Multicolour display and visual temperature detection

The character "8" is fabricated by using **DCzB** powders. Photographs under the excitation of the 365 nm UV light were taken at 300 K, 273 K, 195 K and 77 K respectively to illustrate the potential applications in anti-countefeiting, multicolour display and visual detection of temperature. On the excitation, the "8" pattern shows blue emission at different temperature with negligible color difference. After the cease the excitation, the pattern exhibit obvious color difference from the blue-green afterglow at 300 K to green-yellow long-live emission at 77 K, exhibiting great potential in multicolour display. From the temperature colour chart obtained through the CIE 1931 coordinates

of the afterglow spectra at various temperatures from 77 K to 300 K, the afterglow color gradually changes from green-yellow to blue-green, demonstrating the naked eyes visible detection of temperature in a wide range. The function was fitted for temperature detection based on the Commission International de l'Eclairage (CIE) coordinate diagram of DCzB crystal's afterglow colour under different temperature. Good fitting can be achieved by the fourth order polynomial equation in Supplementary Equation (3) and (4):

$$\mathrm{T} = A_1 + A_2 x + A_3 x^2 + A_4 x^3 + A_5 x^4 \tag{3}$$

$$\mathrm{T} = B_1 + B_2 \mathrm{y} + B_3 y^2 + B_4 y^3 + B_5 y^4 \tag{4}$$

where T represents the temperature from 77 to 300 K. x, y represents the x-coordinate and y-coordinate of afterglow emission colour, respectively. A and B are constants (A_1 = 0.41722, A_2 = -0.00119, A_3 = 1.1422*10^{-5}, A_4 = -3.07067*10^{-8}, A_5 = 3.54869*10^{-12}, R^2 = 0.99; B_1 = 0.55159, B_2 = -2.79942*10^{-5}, B_3 = -3.34644*10^{-7}, B_4 = 1.56719*10^{-8}, B_5 = -5.95813*10^{-11}, R^2 = 0.97). R^2 represents the coefficient of association. Therefore, we can quantitatively identify the visual detection of temperature.

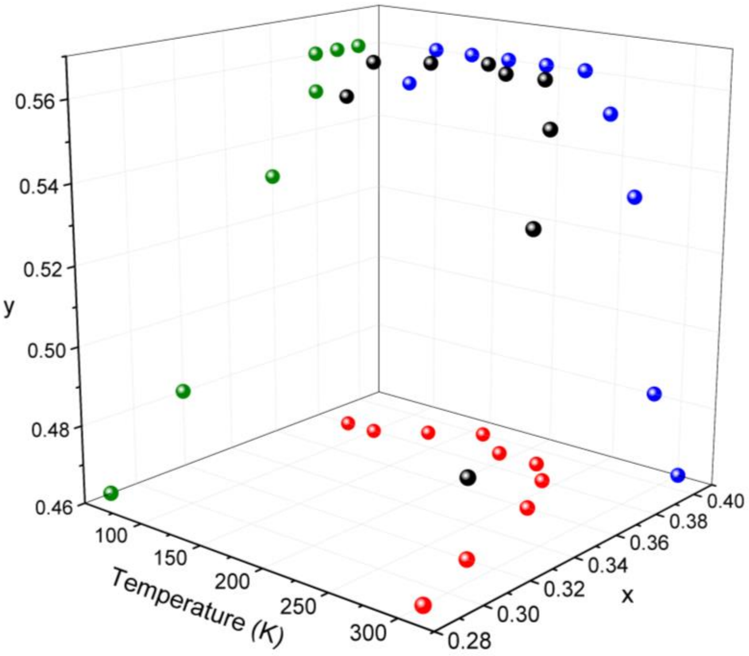

Supplementary Figure 23. The fitting function based on DCzB afterglow emission upon the change of the temperature from 77 to 300 K.

Supplementary References

1. An, Z. et al. Stabilizing triplet excited states for ultralong organic phosphorescence. *Nat. Mater.* **14**, 685-690 (2015).
2. Gong, Y. et al. Achieving Persistent Room Temperature Phosphorescence and Remarkable Mechanochromism from Pure Organic Luminogens. *Adv. Mater.* **27**. 6195-6201 (2015).
3. Zhao, W. et al., Rational Molecular Design for Achieving Persistent and Efficient Pure Organic Room-Temperature Phosphorescence. *Chem.* **1**. 592-602 (2016).
4. Yang, Z. et al. Intermolecular Electronic Coupling of Organic Units for Efficient Persistent Room-Temperature Phosphorescence. *Angew. Chem. Int. Ed.* **55**. 2181-2185 (2016).
5. Xue, P. et al. Bright persistent luminescence from pure organic molecules through a moderate intermolecular heavy atom effect. *Chem. Sci.* **8**. 6060-6065 (2017).
6. Shoji, Y. et al. Unveiling a New Aspect of Simple Arylboronic Esters: Long-Lived Room-Temperature Phosphorescence from Heavy-Atom-Free Molecules. *J. Am. Chem. Soc.* **139**. 2728-2733 (2017).
7. Cai, S. et al. Visible-Light-Excited Ultralong Organic Phosphorescence by Manipulating Intermolecular Interactions. *Adv. Mater.* **29**. 1701244 (2017).
8. Fateminia, S. M. A. et al. Organic Nanocrystals with Bright Red Persistent Room-Temperature Phosphorescence for Biological Applications. *Angew. Chem. Int. Ed.* **56**. 12160-12164 (2017).
9. Xie, Y. et al. How the Molecular Packing Affects the Room Temperature Phosphorescence in Pure Organic Compounds: Ingenious Molecular Design, Detailed Crystal Analysis, and Rational Theoretical Calculations. *Adv. Mater.* **29**. 1606829 (2017).
10. He, Z. et al. White light emission from a single organic molecule with dual phosphorescence at room temperature. *Nat. Commun.* **8**. 416 (2017).
11. Gu, L. et al. Dynamic Ultralong Organic Phosphorescence by Photoactivation. *Angew. Chem. Int. Ed.* **57**. 8425-8431 (2018).
12. Xiong, Y. et al. Designing Efficient and Ultralong Pure Organic Room-Temperature Phosphorescent Materials by Structural Isomerism. *Angew. Chem. Int. Ed.* **57**. 7997-8001 (2018).
13. Tao, Y. et al. Resonance-Activated Spin-Flipping for Efficient Organic Ultralong Room-Temperature Phosphorescence. *Adv. Mater.* **30**. 1803856 (2018).
14. Cai, S. et al. Enhancing Ultralong Organic Phosphorescence by Effective π-Type Halogen Bonding. *Adv. Funct. Mater.* **28**. 1705045 (2018).
15. Chen, B., Zhang, X., Wang, Y., Miao, H. & Zhang, G. Aggregation-Induced Emission with Long-Lived Room-Temperature Phosphorescence from Methylene-Linked Organic Donor-Acceptor Structures. *Chem. - Asian J.* **14**. 751-754 (2019).
16. Shi, H. et al. Highly Efficient Ultralong Organic Phosphorescence through Intramolecular-Space Heavy-Atom Effect. *J. Phys. Chem. Lett.* **10**, 595-600 (2019).
17. Tian, S. et al. Utilizing d-pπ Bonds for Ultralong Organic Phosphorescence. *Angew. Chem. Int. Ed.* **58**. 6645-6649 (2019).
18. Mao, Z. et al. The methylation effect in prolonging the pure organic room temperature phosphorescence lifetime. *Chem. Sci.* **10** 179-184 (2019).
19. Salla, C. A. M. *e*t al. Persistent Solid-State Phosphorescence and Delayed Fluorescence at Room Temperature by a Twisted Hydrocarbon. *Angew. Chem. Int. Ed.* **58**. 6982-6986 (2019).
20. Tu, D. et al. Boron-Cluster-Enhanced Ultralong Organic Phosphorescence. *Angew. Chem. Int. Ed.*

58. 9129-9133 (2019).

21. Li, M. et al. Prolonging Ultralong Organic Phosphorescence Lifetime to 2.5 s through Confining Rotation in Molecular Rotor. *Adv. Opt. Mater.* **7**. 1800820 (2019).
22. He, Z. et al. Achieving Persistent, Efficient, and Robust Room-Temperature Phosphorescence from Pure Organics for Versatile Applications. *Adv. Mater.* **31**. 1807222 (2019).
23. Yuan, J. et al. Direct population of triplet excited states through singlet-triplet transition for visible-light excitable organic afterglow. *Chem. Sci.* **10**. 5031-5038 (2019).
24. Gu, L. et al. Colour-tunable ultra-long organic phosphorescence of a single-component molecular crystal. *Nat. Photon.* **13**. 406-411 (2019).
25. Hirata, S. Roles of Localized Electronic Structures Caused by π Degeneracy Due to Highly Symmetric Heavy Atom-Free Conjugated Molecular Crystals Leading to Efficient Persistent Room-Temperature Phosphorescence. *Adv. Sci.* **6**. 1900410 (2019).
26. Zhao, W. et al. Boosting the efficiency of organic persistent room-temperature phosphorescence by intramolecular triplet-triplet energy transfer. *Nat. Commun.* **10**. 1595 (2019).
27. Yuan, J. et al. Invoking Ultralong Room Temperature Phosphorescence of Purely Organic Compounds through H-Aggregation Engineering. *Mater. Horiz.* **6**. 1259-1264 (2019).
28. Chen, P., Niu, L. Chen, Y. & Yang, Q., Difluoroboron β-diketonate dyes: Spectroscopic properties and applications. *Coord. Chem. Rev.* **350**. 196-216 (2017).
29. Li, D., Zhang, H. & Wang, Y. Four-coordinate organoboron compounds for organic light-emitting diodes (OLEDs). *Chem. Soc. Rev.* **42**. 8416-8433 (2013).
30. Chen, P. et al. A Solid-State Fluorescent Material Based on Carbazole-Containing Difluoroboron β-Diketonate: Multiple Chromisms, the Self-Assembly Behavior, and Optical Waveguides. *Adv. Funct. Mater.* **27**. 1700332 (2017).
31. Ahn, D. H. et al. Highly efficient blue thermally activated delayed fluorescence emitters based on symmetrical and rigid oxygen-bridged boron acceptors. *Nat. Photon.* **13**. 540-546 (2019).
32. Tao, Y. et al. Resonance-Activated Spin-Flipping for Efficient Organic Ultralong Room-Temperature Phosphorescence. *Adv. Mater.* **30**. 1803856 (2018).
33. Baryshnikov, G., Minaev, B. & Ågren, H. Theory and Calculation of the Phosphorescence Phenomenon. *Chem. Rev.* **117**. 6500-6537 (2017).
34. Aidas, K. et al. The Dalton quantum chemistry program system. *Wiley Interdiscip. Rev.: Comput. Mol. Sci.* **4**. 269-284 (2014).
35. An, Z. et al. Stabilizing triplet excited states for ultralong organic phosphorescence. *Nat. Mater.* **14**, 685-690 (2015).
36. Kasha, M. Rawls, H. & Ashraf El-Bayoumi, M. The exciton model in molecular spectroscopy. *Pure Appl. Chem.* **11**. 371-392 (1965).
